# Immunosenescence: How Aging Increases Susceptibility to Bacterial Infections and Virulence Factors

**DOI:** 10.3390/microorganisms12102052

**Published:** 2024-10-11

**Authors:** Nikolaos Theodorakis, Georgios Feretzakis, Christos Hitas, Magdalini Kreouzi, Sofia Kalantzi, Aikaterini Spyridaki, Zoi Kollia, Vassilios S. Verykios, Maria Nikolaou

**Affiliations:** 1 Department of Cardiology, Amalia Fleming General Hospital, 14, 25th Martiou Str., 15127 Melissia, Greece; n.theodorakis@flemig-hospital.gr (N.T.); ch.chitas@flemig-hospital.gr (C.H.); m.nikolaou@flemig-hospital.gr (M.N.); 2 65+ Clinic, Amalia Fleming General Hospital, 14, 25th Martiou Str., 15127 Melissia, Greece; kreouzi.m@live.unic.ac.cy (M.K.); sofia_kalanji@yahoo.gr (S.K.); kspyridaki@yahoo.gr (A.S.); zoi.kollia@yahoo.gr (Z.K.); 3 School of Medicine, National and Kapodistrian University of Athens, 75 Mikras Asias, 11527 Athens, Greece; 4 School of Science and Technology, Hellenic Open University, 18 Aristotelous Str., 26335 Patras, Greece; verykios@eap.gr; 5 Department of Internal Medicine, Amalia Fleming General Hospital, 14, 25th Martiou Str., 15127 Melissia, Greece

**Keywords:** immunosenescence, inflammaging, immune response, bacterial infections, virulence factors, machine learning, aging, elderly

## Abstract

The process of aging leads to a progressive decline in the immune system function, known as immunosenescence, which compromises both innate and adaptive responses. This includes impairments in phagocytosis and decreased production, activation, and function of T- and B-lymphocytes, among other effects. Bacteria exploit immunosenescence by using various virulence factors to evade the host’s defenses, leading to severe and often life-threatening infections. This manuscript explores the complex relationship between immunosenescence and bacterial virulence, focusing on the underlying mechanisms that increase vulnerability to bacterial infections in the elderly. Additionally, it discusses how machine learning methods can provide accurate modeling of interactions between the weakened immune system and bacterial virulence mechanisms, guiding the development of personalized interventions. The development of vaccines, novel antibiotics, and antivirulence therapies for multidrug-resistant bacteria, as well as the investigation of potential immune-boosting therapies, are promising strategies in this field. Future research should focus on how machine learning approaches can be integrated with immunological, microbiological, and clinical data to develop personalized interventions that improve outcomes for bacterial infections in the growing elderly population.

## 1. Introduction

Population aging is a global phenomenon affecting most of our developed societies. Geriatric patients are marked by unique characteristics, including frailty, sarcopenia, malnutrition, cognitive impairment, multimorbidity, and polypharmacy [1]. These factors, together with cellular and molecular changes due to aging, result in a progressive decline in immune function, known as immunosenescence [2]. Furthermore, elderly patients have twice the risk of hospitalization compared to middle-aged patients, which increases the risk of colonization by multidrug-resistant (MDR) bacteria [3]. All of the above factors lead to increased susceptibility to bacterial infections, complications, prolonged hospital stay lengths, and higher mortality rates. According to Eurostat data for 2012, hospitalization rates for infections show a progressive increase with age, ranging from 126 per 100,000 in ages 40–44, tripling to 438.9 per 100,000 in ages 65–69, and further increasing fourfold to 1238.3 per 100,000 in patients aged 80–84 years [4]. Mortality rates also increase with age. In Greece in 2020, the mortality rate due to infections was 40.1 per 100,000 in patients aged 65–79 years, while in patients aged 80+, it was five times higher (389.8 per 100,000) [5].

Elderly individuals are particularly vulnerable to various bacterial infections, with urinary tract infections (UTIs) and respiratory tract infections being the most common. UTIs are frequently caused by *Enterobacterales* (e.g., *Escherichia coli*, *Klebsiella pneumoniae*), *Pseudomonas aeruginosa*, *Acinetobacter baumannii*, and *Enterococcus* spp. Similarly, respiratory infections often involve pathogens such as *Streptococcus pneumoniae*, *Moraxella catarrhalis*, *Haemophilus influenzae*, *Enterobacterales*, as well as *P. aeruginosa* and *A. baumannii* [3,6,7].

In addition, the elderly are prone to skin and soft tissue infections, often linked to *Streptococcus pyogenes* and *Staphylococcus aureus*, as well as infections like pseudomembranous colitis, caused by *Clostridioides difficile*. Bloodstream infections, infective endocarditis (frequently caused by *S. aureus*, *Streptococci viridans*, and *Enterococcus* spp.), and central nervous system infections (commonly due to *S. pneumoniae*, *Neisseria meningitidis*, *H. influenzae*, and *Listeria monocytogenes*) are also prevalent [6,7].

Bacterial infections in this population are categorized as either community-acquired or hospital-acquired, with the latter typically occurring 48 h after hospital admission. Hospital-acquired infections often involve multidrug-resistant (MDR) pathogens, including Gram-negative bacteria like *Enterobacterales*, *P. aeruginosa*, and *A. baumannii*, as well as Gram-positive organisms like methicillin-resistant *S. aureus* (MRSA) and vancomycin-resistant *Enterococcus* spp. (VRE) [6,7,8,9]. The rise in MDR pathogens is largely driven by antibiotic overuse and misuse.

**Figure 1:**
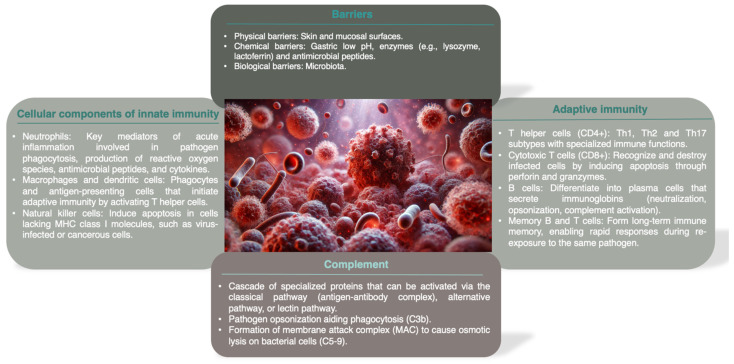
Summary of the components of the immune system.

The immune system, divided into innate and adaptive components, is responsible for combating infections caused by pathogens and maintaining homeostasis [10]. A summary of the components of the immune system is illustrated in Figure 1.

Bacteria utilize a variety of mechanisms to survive in their environments, some of which contribute directly to disease in the host, while others enhance their ability to thrive and persist. It is important to differentiate between virulence factors, which directly promote disease, and fitness factors (e.g., bacterial cell wall), which support bacterial survival but may incidentally contribute to pathogenesis. Some fitness factors (e.g., cell wall) are not virulence factors per se, but play an important role in the pathogenesis of diseases by activating the immune system and potentially leading to severe dysregulated responses and sepsis [11,12].

With advancing age, there is a decline in immune function, known as immunosenescence. The main objective of this review is to explore the complex relationship between immunosenescence and bacterial virulence, focusing on the underlying mechanisms of increased vulnerability to the most frequent MDR bacteria that affect the elderly and summarizing potential solutions. Furthermore, there is a discussion on how machine learning (ML) technologies can provide deeper insights into bacterial virulence and immune responses, potentially leading to personalized medical interventions. 

## 2. Immunosenescence: Aging and the Immune System

As already stated, immunosenescence refers to the gradual decline in immune function associated with aging, characterized by both quantitative and qualitative impairments in innate and adaptive immune responses. This age-related deterioration leads to increased susceptibility to infections, diminished vaccine efficacy, and a heightened risk of chronic inflammatory conditions. At the core of immunosenescence is the accumulation of senescent immune cells, which secrete senescence-associated secretory phenotype (SASP) factors that contribute to systemic inflammation, often termed inflammaging. This chronic, low-grade inflammatory environment exacerbates tissue damage, impairs immune regulation, and drives the progression of age-related diseases [13,14,15].

### 2.1. Thymic Involution and T Cell Dysfunction

One of the most significant age-related changes in the immune system is thymic involution, a process in which the thymus gradually atrophies, leading to a drastic reduction in the output of naïve T cells. This loss of thymic function diminishes the diversity of the TCR repertoire, reducing the immune system’s ability to respond to novel antigens. Consequently, elderly individuals exhibit a reduced ability to mount robust immune responses against newly encountered pathogens, including viruses, bacteria, and tumors. The T cell population becomes dominated by memory T cells, many of which adopt a senescent phenotype, marked by a loss of proliferative capacity and the downregulation of critical co-stimulatory molecules like CD28. Senescent T cells, especially CD8+ T cells, also contribute to the SASP by secreting pro-inflammatory cytokines such as IL-6 and TNF-α, further promoting a pro-inflammatory environment that accelerates immune dysfunction [13,14,15,16]. 

Senescent T cells are often exhausted, characterized by impaired effector functions and the upregulation of inhibitory receptors such as PD-1 and CTLA-4, limiting their cytotoxic activity and ability to clear infections or malignant cells. Thymic involution not only decreases immune competence but also plays a central role in age-related vulnerability to diseases. Over time, the immune system’s inability to generate sufficient numbers of naive T cells and the increasing dominance of dysfunctional memory T cells contribute to an overall decline in adaptive immune responses [13,14,15,16]. 

### 2.2. Impact on T Helper Cells

Aging also affects T helper (CD4+) cell function, specifically disrupting the balance between Th1 and Th2 responses. There is a notable shift towards Th2 dominance, which skews immune responses toward humoral immunity at the expense of effective cellular responses. Th1 cells, which are essential for activating macrophages and clearing intracellular pathogens, become functionally impaired, contributing to an increased susceptibility to intracellular infections such as *Mycobacterium tuberculosis*. While the dominance of Th2 responses enhances the body’s ability to fight certain extracellular pathogens through increased antibody levels, it may paradoxically leave individuals more vulnerable to specific pathogens capable of evading or resisting the humoral immune response, such as *S. aureus*. In addition, the regulatory T cell (Treg) compartment also undergoes age-related changes, with Tregs showing reduced suppressive function and impaired control over immune activation, potentially contributing to autoimmunity and chronic inflammation during aging [13,14,15,17].

### 2.3. B Cell Dysfunction

The aging process profoundly impacts B cell function, leading to a reduction in the generation of naïve B cells due to a decline in hematopoietic stem cell function in the bone marrow. As a result, the B cell population becomes skewed towards memory B cells, many of which exhibit impaired responsiveness and reduced capacity for class switch recombination (CSR), a process critical for the production of high-affinity, isotype-switched antibodies (e.g., IgG). Additionally, aging B cells produce lower levels of protective antibodies following infection or vaccination, contributing to weaker immune responses in elderly individuals. The inability to mount robust antibody responses is one of the key reasons for the decreased efficacy of vaccines such as the influenza and pneumococcal vaccines in older populations. The diminished capacity for somatic hypermutation (SHM) and CSR in aging B cells results in antibodies that are lower in affinity and less capable of neutralizing pathogens. This is compounded by a pro-inflammatory environment driven by senescent immune cells, which produce high levels of cytokines such as IL-6 and TNF-α, further impairing B cell function and contributing to overall immune decline [13,14,15,18].

### 2.4. Senescence-Associated Secretory Phenotype (SASP) and Inflammaging

SASP is a hallmark of senescent cells, including senescent immune cells. SASP refers to the secretion of a wide array of pro-inflammatory factors, including IL-6, IL-1β, TNF-α, and matrix metalloproteinases (MMPs), that perpetuate chronic inflammation. In the context of immunosenescence, SASP drives a state of chronic, low-grade systemic inflammation, termed inflammaging. This persistent inflammatory milieu not only contributes to the deterioration of immune function but also promotes the onset and progression of age-related diseases such as cardiovascular disease, neurodegenerative disorders, and metabolic syndrome. SASP factors exacerbate tissue damage by promoting extracellular matrix degradation, facilitating the infiltration of immune cells into tissues, and driving the development of fibrosis. Moreover, the interplay between SASP and chronic immune activation accelerates the decline of immune competence [13,14,15,19].

### 2.5. Innate Immune Dysfunction

Aging affects multiple components of the innate immune system, including neutrophils, macrophages, and dendritic cells. Neutrophils exhibit reduced chemotactic activity, impaired phagocytosis, and decreased production of ROS, all of which are critical for the clearance of bacterial pathogens. These functional impairments diminish the ability of neutrophils to mount effective responses against infections, contributing to increased vulnerability in older adults. Similarly, macrophages in aged individuals show diminished phagocytic capacity and impaired antigen presentation, which hampers the initiation of adaptive immune responses by T cells. Aging macrophages also produce altered levels of cytokines, which may exacerbate tissue damage during inflammation. Dendritic cells, the key antigen-presenting cells that initiate T cell activation, also exhibit reduced functionality with age, including decreased ability to capture, process, and present antigens to naive T cells. These changes collectively weaken the immune system’s ability to respond effectively to pathogens, compounding the effects of immunosenescence [13,14,15,20].

### 2.6. Vaccine Efficacy and Immunosenescence

The decline in both innate and adaptive immune function with aging directly impacts the efficacy of vaccines. Vaccines rely on the generation of robust T and B cell responses, both of which are impaired in the elderly due to reduced production of naive immune cells, impaired memory cell function, and diminished antibody responses. Consequently, elderly individuals often exhibit lower antibody titers and shorter-lasting immunity following vaccination, as seen with the influenza and pneumococcal vaccines. The impaired functionality of B cells and reduced capacity to generate high-affinity antibodies and memory T cells result in weaker and less durable immune protection in older populations. To mitigate these effects, vaccines specifically designed for the elderly, such as adjuvanted or high-dose vaccines, have been developed to enhance immune responses and provide better protection. These modified vaccines aim to compensate for the age-related decline in immune competence by boosting the activation of immune cells and promoting stronger immunogenicity [13,14,15,21].

An overview of the mechanisms of immunosenescence and the key bacteria involved is presented in Table 1.

**Figure 2:**
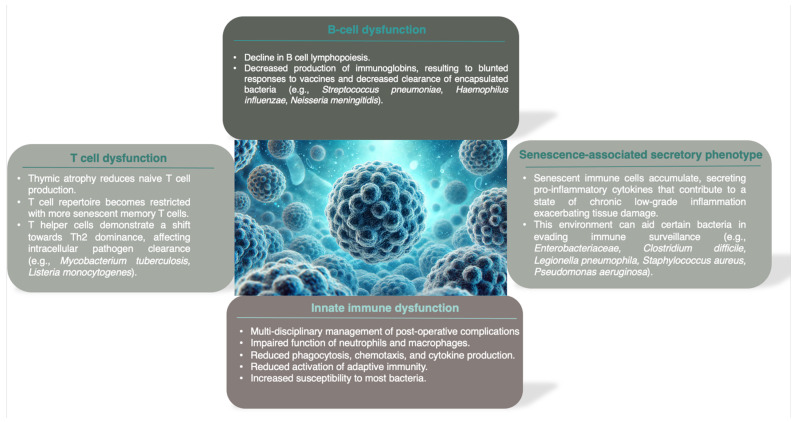
Key features of immunosenescence.

The key features of immunosenescence are illustrated in Figure 2.

## 3. Interaction between Immunosenescence and the Most Common MDR Bacteria Affecting the Elderly

While elderly individuals are more susceptible to bacterial infections due to immunosenescence, it is important to consider that many infections commonly seen in older adults—such as those caused by *C. difficile*, VRE, and *A. baumannii*—are not exclusively a result of aging or immune decline. Instead, these infections are often associated with additional factors such as frequent hospitalizations, increased use of invasive medical devices (e.g., catheters, ventilators), and the higher prevalence of comorbidities like cardiovascular disease, cancer, and diabetes, which can lead to immunosuppression [3]. 

For example, MDR bacteria tend to be more prevalent in hospital settings, where older adults are frequently admitted for the management of chronic conditions. This increased exposure to healthcare environments, along with factors like recent antibiotic use, plays a critical role in the higher incidence of these infections in elderly populations. Therefore, while immunosenescence contributes to increased susceptibility, the occurrence of infections caused by MDR bacteria in the elderly must be viewed within the broader context of hospitalization and comorbidities, rather than as a direct consequence of aging alone [3]. 

A recent study with data from long-term care facilities worldwide allows us to estimate the most common MDR bacteria affecting the elderly [22]. Based on the findings of this study, we will focus our discussion on MRSA, Enterobacterales (especially *E. coli* and *K. pneumoniae*), *P. aeruginosa*, and *A. baumannii*.

### 3.1. Mucosal Barriers 

Mucociliary clearance is a crucial defense mechanism in the respiratory tract, where the mucociliary escalator traps inhaled particles, including pathogens, in mucus and removes them through coordinated ciliary movement. In elderly individuals, this system becomes less effective due to a decline in ciliary function and reduced mucus production. This makes it harder to expel mucus along with trapped pathogens, providing an opportunity for bacteria such as *P. aeruginosa*, *K. pneumoniae*, *A. baumannii*, and MRSA to colonize the respiratory tract. This is especially problematic in bedridden or ventilated patients, where immobility further exacerbates mucus stasis. The result is an environment where these pathogens, which are often opportunistic and MDR, can thrive, leading to severe pneumonia. This also hinders the elimination of pathogens after infection, leading to persistency, extended hospital stays, and increased risk of complications, allowing them to establish persistent infections [23].

Secretory IgA (sIgA) is a critical component of mucosal immunity, particularly in defending against bacterial and viral pathogens at mucosal surfaces, such as the respiratory, gastrointestinal, and urinary tracts. sIgA functions by neutralizing pathogens before they can adhere to epithelial cells, preventing colonization and invasion. In elderly individuals, the levels of sIgA decrease significantly, leaving mucosal surfaces more vulnerable to infection. This decline in sIgA is particularly concerning for pathogens like *Enterobacterales*, MRSA, *P. aeruginosa*, and *A. baumannii*, which are known for colonizing compromised mucosal surfaces. Without sufficient sIgA, these bacteria can more easily adhere to and penetrate mucosal barriers, leading to severe and persistent infections, including pneumonia and UTIs [24]. 

### 3.2. Biofilm Formation

Biofilm formation is a critical virulence factor, particularly in healthcare settings where elderly patients frequently require invasive medical devices like catheters, ventilators, and prosthetic joints. In these environments, bacteria form biofilms—complex, structured communities of cells encased in a self-produced extracellular matrix composed of polysaccharides, proteins, lipids, and extracellular DNA. 

For MRSA, biofilm formation is particularly problematic on surfaces such as indwelling catheters and prosthetic joints. MRSA biofilms are highly resistant to both the host immune system and antibiotic therapies, owing to the slow growth of bacteria in the deeper layers of the biofilm and the limited penetration of drugs through the matrix. This defensive structure helps MRSA evade phagocytosis and the bactericidal action of immune cells like neutrophils. The persistent presence of biofilms can lead to chronic infections, particularly in elderly individuals whose immune systems are less effective at mounting responses to clear these infections, resulting in prolonged and difficult-to-treat infections, particularly in the bloodstream and soft tissues surrounding medical devices [25]. 

*P. aeruginosa* and *A. baumannii* are also notorious for biofilm formation, especially in hospital environments where they colonize ventilators and catheters, leading to ventilator-associated pneumonia (VAP) and catheter-associated UTIs. *P. aeruginosa* is a particularly formidable biofilm-former, known for its thick matrix composed of alginate, which not only provides protection from antibiotics but also shields the bacteria from oxidative stress and host immune responses. This pathogen is a major cause of chronic respiratory infections, particularly in immunocompromised and elderly patients, especially those with chronic obstructive pulmonary disease or those on mechanical ventilation. Similarly, *A. baumannii*, which thrives in ICU settings, forms biofilms on medical devices and surfaces, contributing to its resistance to antibiotics and environmental disinfectants. Biofilm-associated infections caused by these pathogens are difficult to eradicate because the bacteria in biofilms exhibit significantly reduced metabolic activity, making them less susceptible to antibiotics that target actively dividing cells. Additionally, the matrix physically blocks immune cells like macrophages and neutrophils from reaching and killing the bacteria. The result is a cycle of chronic infections that require prolonged antibiotic therapy, often with limited success [26,27]. 

Biofilm formation by *E. coli* and *K. pneumoniae* is a key factor in recurrent UTIs and pneumonia, particularly in elderly patients who are frequently catheterized or ventilated. Once adhered to the uroepithelial cells, *E. coli* forms biofilms that protect the bacteria from the flushing action of urine and immune surveillance, allowing them to persist and cause recurrent infections. Similarly, *K. pneumoniae* forms biofilms in both the lungs and on medical devices, such as ventilators, where it causes severe respiratory infections. Biofilms of *K. pneumoniae* are rich in capsular polysaccharides that not only inhibit the penetration of antibiotics but also interfere with the ability of the immune system to clear the infection. The formation of these biofilms, particularly in the context of MDR strains, is associated with significant morbidity and mortality in hospitalized elderly patients, as they contribute to persistent, hard-to-treat infections that can lead to sepsis, serious complications, and extended hospital stays [28,29].

### 3.3. Impaired Chemotaxis of Phagocytes and Neutrophils

One of the key issues is the age-related decline in chemotaxis, the process by which immune cells are directed to infection sites via chemokine signaling. Chemokine receptors like *CXCR1* and *CXCR2*, which play a crucial role in guiding neutrophils and macrophages, are downregulated in the elderly. This impairs the migration of immune cells, delaying their arrival at infection sites, which allows bacteria to establish biofilms more easily. For example, *P. aeruginosa* and *A. baumannii* take advantage of this immune delay to form biofilms that are difficult to eradicate. Similarly, *K. pneumoniae* and *E. coli* can establish persistent UTIs and respiratory infections in elderly patients, as the reduced immune response allows these bacteria to adhere, colonize, and form protective biofilm communities [13,14,15,30]. 

Once neutrophils and macrophages do reach the site of infection, their ability to penetrate biofilms and exert their bactericidal functions is further compromised. Neutrophils, which rely on the NADPH oxidase complex to produce reactive oxygen species (ROS) and on inducible nitric oxide synthase (iNOS) to generate nitric oxide (NO), are less effective in elderly individuals. These reactive molecules are essential for killing bacteria within biofilms, but in the elderly, their production is significantly reduced, allowing biofilm-encased bacteria like MRSA, *P. aeruginosa*, and *A. baumannii* to persist [13,14,15,20]. 

In the case of MRSA, mechanisms of resistance to phagocytes are even more formidable. This pathogen has evolved strategies to degrade neutrophil extracellular traps (NETs)—web-like structures released by neutrophils during a form of cell death called NETosis—by expressing DNases that break down these antimicrobial DNA-protein complexes. Age-related defects in intracellular signaling pathways and chromatin decondensation also impair NET formation in the elderly, further reducing the ability to contain and kill MRSA within biofilms [31,32]. In addition to biofilm resilience, MRSA is recognized by Toll-like receptor 2 (TLR2), which detects bacterial components like lipoteichoic acids and peptidoglycans. However, in the elderly, TLR2 signaling through NF-κB is often dysregulated, resulting in delayed and reduced production of pro-inflammatory cytokines like TNF-α and IL-6. This attenuates the recruitment and activation of immune cells, which are critical for mounting a robust response to MRSA infections [33]. Consequently, biofilm-associated infections persist longer and are more resistant to clearance. 

### 3.4. Impaired T and B Cell Function 

As already analyzed in the previous section, the senescence of adaptive immunity, particularly the decline in T and B cell functionality, has profound effects on the immune system’s ability to respond to bacterial infections. As individuals age, thymic involution leads to a significant reduction in the production of naive T cells, which are essential for mounting adaptive immune responses against new pathogens. In the case of MRSA, the decreased number and functionality of Th1 and Th17 cells are especially detrimental. Th1 cells, which produce interferon-gamma (IFN-γ), are critical for activating macrophages and enhancing their bactericidal activity. However, with age, IFN-γ production declines, resulting in less effective macrophage activation and reduced intracellular killing of MRSA, which is notorious for surviving within host cells [13,14,15,16]. Th17 cells, which are responsible for producing IL-17, play a key role in recruiting and activating neutrophils, the primary cells responsible for killing extracellular bacteria. The age-related reduction in IL-17 production further diminishes the neutrophil response, making it harder to clear MRSA and other biofilm-forming pathogens like *P. aeruginosa* and *A. baumannii*. This impaired T cell function allows these bacteria to persist and form chronic, biofilm-associated infections, particularly on medical devices commonly used in elderly patients, such as catheters and ventilators [34]. 

The decline in B cell function with aging compounds the problem, particularly in combating MRSA and other encapsulated bacteria like *K. pneumoniae* and *E. coli*. B cell senescence is characterized by a reduced ability to undergo affinity maturation and class-switch recombination, processes that are crucial for generating high-affinity, class-switched antibodies such as IgG. These antibodies are essential for opsonizing bacteria, marking them for phagocytosis by neutrophils and macrophages [13,14,15,18]. In the case of MRSA, which expresses protein A to block Fcγ receptor-mediated opsonization, the immune system relies heavily on the production of high-affinity antibodies to counteract this defense mechanism. In elderly individuals, however, the reduced generation of high-affinity IgG antibodies significantly weakens opsonization, allowing MRSA to evade immune clearance [35]. Furthermore, aged B cells exhibit diminished memory B cell formation, leading to a reduced capacity to mount a rapid and effective secondary immune response upon reinfection. This is particularly problematic in recurrent infections caused by biofilm-forming bacteria like *E. coli* and *K. pneumoniae* [13,14,15,18]. 

### 3.5. Notable Virulence Factors

One of MRSA’s key virulence factors is protein A, a cell wall-anchored protein that binds the Fc region of IgG antibodies in reverse. Normally, IgG coats bacteria through a process called opsonization, making them recognizable to immune cells like neutrophils and macrophages, which have Fcγ receptors (FcγRs) that bind to the Fc region of IgG. However, protein A disrupts this process by binding IgG itself, preventing immune cells from detecting and phagocytosing MRSA. This immune evasion is particularly dangerous in elderly patients, whose neutrophils and macrophages are already compromised due to reduced chemotaxis, phagocytic activity, and decreased ROS production. With fewer bacteria being engulfed and killed, MRSA persists and multiplies, leading to chronic or recurrent infections, particularly in biofilm-associated infections involving medical devices, where MRSA is further shielded from immune surveillance and antibiotics [35,36]. 

Both *E. coli* and *K. pneumoniae* use fimbrial adhesins as a primary virulence factor to colonize epithelial surfaces. *E. coli* expresses type 1 fimbriae and P fimbriae, which facilitate adherence to uroepithelial cells, a crucial step in the development of UTIs. *K. pneumoniae* utilizes similar fimbrial adhesins to adhere to respiratory epithelial cells, promoting lung infections like pneumonia. Once adhesion is established, these pathogens can invade deeper tissues. In elderly individuals, epithelial integrity is already compromised due to weakened tight junctions and diminished expression of junctional proteins like claudins and occludins, which normally maintain the barrier between epithelial cells. This weakening allows for easier invasion by *E. coli* and *K. pneumoniae*, enabling these pathogens to penetrate deeper tissues and establish infections more readily [37,38]. 

*A. baumannii* expresses several virulence factors, but Outer Membrane Protein A (*OmpA*) is particularly significant in mediating immune evasion and pathogenesis. *OmpA* facilitates bacterial adherence to host epithelial cells and promotes biofilm formation on medical devices like catheters and ventilators. Moreover, *OmpA* inhibits the fusion of phagosomes with lysosomes inside phagocytes, such as neutrophils and macrophages, thereby preventing the bacteria from being destroyed within these immune cells. *OmpA* also induces apoptosis of phagocytes and epithelial cells, leading to disruption of epithelial barriers and promoting systemic dissemination of the pathogen. In elderly individuals, who already suffer from impaired neutrophil and macrophage function, as well as slower tissue repair mechanisms, *OmpA*’s ability to induce cell death and disrupt barriers leads to more widespread and difficult-to-control infections [39]. 

*P. aeruginosa* is equipped with an array of potent virulence factors that contribute to severe tissue damage and immune evasion, especially in elderly patients with weakened immune systems. One of its most lethal virulence factors is Exotoxin A (ETA), which inhibits host protein synthesis by ADP-ribosylating elongation factor 2 (EF-2). This halts protein synthesis in host cells, leading to widespread cell death and tissue necrosis. In the lungs, ETA can cause severe respiratory epithelial damage, impairing mucociliary clearance, which is already reduced in elderly individuals. This makes it easier for *P. aeruginosa* to establish chronic lung infections, such as VAP. In addition, *P. aeruginosa* produces pyocyanin, a redox-active virulence factor that generates ROS, causing oxidative stress and damage to host cells. As already analyzed, elderly individuals often have diminished antioxidant defenses, amplifying the oxidative damage caused by pyocyanin, which compromises both epithelial and immune cell function. This leads to increased tissue injury and impaired immune responses. The bacterium also produces elastases (LasA and LasB), proteases that degrade host tissues, extracellular matrix components, and immune proteins like complement and immunoglobulins. These elastases promote bacterial dissemination and impair the immune response, further contributing to chronic, hard-to-treat infections in elderly patients [40]. 

In addition to fimbrial adhesins, *E. coli* and *K. pneumoniae* produce capsular polysaccharides that help these bacteria evade immune responses. The capsule forms a physical barrier that protects the bacteria from being phagocytosed by immune cells and helps them resist complement-mediated lysis. This is particularly problematic in elderly individuals, whose immune systems are already less efficient at producing high-affinity antibodies and recruiting immune cells to infection sites. *K. pneumoniae*’s thick polysaccharide capsule is especially adept at preventing opsonization and phagocytosis, allowing the bacteria to persist in the lungs or urinary tract and cause prolonged infections. *E. coli*, particularly uropathogenic strains, also produces toxins like hemolysin, which can lyse host cells and contribute to tissue damage in the urinary tract, leading to more severe and recurrent UTIs in elderly individuals [41,42]. 

### 3.6. Siderophores

Iron is a critical nutrient for bacterial survival and replication, yet free iron is limited within the host due to a mechanism called “nutritional immunity”. Host proteins, such as lactoferrin and hepcidin, tightly regulate iron availability to prevent bacterial growth. However, many bacteria have evolved to overcome this restriction through the production of siderophores (iron-chelating molecules) [43]. 

*P. aeruginosa* produces siderophores such as pyoverdine and pyochelin, *A. baumannii* produces acinetobactin, while *Enterobacterales* like *E. coli* and *K. pneumoniae* produce enterobactin and aerobactin. These siderophores have a high affinity for iron and can outcompete host proteins, allowing the bacteria to acquire sufficient iron for survival and replication. In the elderly, the dysregulation of iron metabolism becomes even more problematic [43,44]. Pyoverdine is *P. aeruginosa*’s primary siderophore and one of the most potent iron-chelating molecules it produces. Pyoverdine has a high affinity for ferric iron (Fe^3+^), and it not only scavenges iron from the host’s iron-binding proteins like transferrin and lactoferrin but also acts as a signaling molecule, regulating the expression of various virulence factors. The production of pyoverdine enables *P. aeruginosa* to survive and proliferate in nutrient-limited environments such as the lungs during chronic infections, like those seen in cystic fibrosis or VAP. Though less efficient than pyoverdine in terms of iron affinity, pyochelin complements *P. aeruginosa*’s iron acquisition strategy by binding iron in slightly different conditions where pyoverdine may be less effective. Pyochelin plays an important role in establishing and maintaining infections, particularly in biofilms where bacterial access to iron can be limited [44].

Acinetobactin is the key siderophore for *A. baumannii* and plays a critical role in its ability to cause infections, especially in nutrient-depleted environments such as medical devices or within host tissues. Acinetobactin has a high affinity for iron and allows A. baumannii to effectively compete for iron even when it is tightly bound by host proteins. This siderophore is especially critical for *A. baumannii*’s survival in hospital environments, where it is frequently responsible for multidrug-resistant infections, such as VAP and catheter-related infections [45].

Enterobactin is one of the most efficient siderophores produced by *E. coli and K. pneumoniae*, with an extremely high affinity for ferric iron (Fe^3+^). Enterobactin can outcompete host iron-sequestering proteins like transferrin and lactoferrin, making it a powerful tool for iron acquisition in iron-limited environments, such as UTIs or pneumonia. The ability of enterobactin to bind iron is a key factor in the pathogenicity of *E. coli* and *K. pneumoniae*, allowing them to establish and maintain infections even when iron availability is low [46].

Aerobactin is particularly important for pathogenic strains of *E. coli* and *K. pneumoniae* that cause extraintestinal infections. While enterobactin is more efficient at binding iron, aerobactin can acquire iron in different environments, particularly when the host’s immune response limits the availability of iron. This versatility makes *E. coli* and *K. pneumoniae* highly adaptable and capable of causing a broad range of infections, including bloodstream infections and pneumonia, especially in elderly and immunocompromised individuals [47].

In the context of aging and immunosenescence, the production of these siderophores becomes even more dangerous. Specifically, the acute-phase response, which would typically bolster iron sequestration during infection, is blunted in older adults. This allows iron to remain more available to pathogens in nutrient-limited environments like the urinary tract and lungs [13,14,15]. 

## 4. Clinical Implications for Prevention and Management of Bacterial Infections and the Role of ML

### 4.1. Vaccinations

Vaccinations play a critical role in preventing bacterial infections in elderly populations, though their efficacy can be compromised by immunosenescence and bacterial virulence mechanisms. Immunosenescence results in a reduced production of naïve T cells, impaired antigen presentation, and altered cytokine responses, all of which decrease vaccine effectiveness in older adults. Additionally, bacterial virulence factors, such as biofilm formation and immune evasion strategies, further complicate the efficacy of vaccines [13,14,15,21]. 

Currently, vaccines targeting *S. pneumoniae*—a leading cause of pneumonia in the elderly—focus on pneumococcal capsular polysaccharides. However, due to immunosenescence, vaccine efficacy diminishes, prompting ongoing research into enhanced formulations. Beyond *S. pneumoniae*, there is growing interest in vaccines targeting *C. difficile* and *K. pneumoniae*, both of which significantly affect elderly patients. For example, a toxin-based vaccine for *C. difficile* showed limited benefits in clinical trials [48], while vaccines against *K. pneumoniae* are being explored, focusing on capsular polysaccharides and siderophores such as enterobactin to prevent infections like UTIs and pneumonia [49].

ML can play a potential role in vaccine development by accelerating the identification of B and T cell epitopes, which are key targets for immune responses. Traditional epitope identification methods are laborious, but ML allows the analysis of large-scale data to streamline this process. For example, ML-based reverse vaccinology pipelines like *NetMHCpan* have been used to predict T cell epitopes by analyzing peptide-HLA binding data. In recent studies, models like MHCflurry (OpenVax, Hammer Lab, New York, NY, USA) and NetMHCpan (Technical University of Denmark (DTU) Bioinformatics, Lyngby, Denmark) achieved high predictive accuracy (Area Under the Curve [AUC] scores of 0.85 and above) in identifying presented epitopes in large datasets, such as those available in the Immune Epitope Database (IEDB). These models are trained on thousands of experimentally validated peptide-HLA binding pairs, enabling rapid selection of potential vaccine targets, as demonstrated in the development of SARS-CoV-2 vaccines. By using such models, researchers can focus on the most immunogenic candidates, significantly reducing the time and cost of epitope identification [50,51,52].

In addition to epitope identification, ML can be potentially used to predict the immunogenicity of selected vaccine targets. This involves predicting how effectively a selected epitope will trigger immune responses, including antibody and T cell receptor binding. Supervised learning algorithms, such as deep neural networks, have been employed to predict B cell epitopes with an average AUC score of ~0.8. For example, deep learning models trained on structural data from protein sequences have improved the prediction of conformational B cell epitopes by incorporating features like solvent accessibility, residue flexibility, and evolutionary conservation. These models, such as AlphaFold (DeepMind, London, UK) and trRosetta (Baker Lab, University of Washington, Seattle, WA, USA), predict how protein structures fold and interact, allowing researchers to identify which epitopes are likely to be presented on the pathogen surface and recognized by the immune system. In one recent application, AlphaFold predicted the structures of over 350,000 proteins, which could have implications for improving vaccine antigen selection based on structural insights [50,51,52].

Moreover, ML could assist in optimizing vaccine adjuvants and formulations. By analyzing clinical trial data, ML models like autoencoders and supervised learning algorithms have been used to identify optimal adjuvant formulations that boost immune responses in specific populations. For instance, autoencoders have been applied to predict immune cell activation patterns, which correlate with successful vaccine responses in elderly populations with immunosenescence. In one study, supervised learning models were used to predict vaccine adjuvant efficacy, with accuracy rates exceeding 75%. Additionally, ML models like AlphaFold (DeepMind, London, UK) and RoseTTAFold (Baker Lab, University of Washington, Seattle, WA, USA) can predict mRNA stability and protein folding, which is crucial for the design of mRNA vaccines. These models help optimize vaccine design by predicting how mRNA or protein antigens will behave in the host, allowing for better-targeted and more durable vaccines, as evidenced by their application in the development of COVID-19 mRNA vaccines [50,51,52].

### 4.2. Immune Rejuvenation

Immune rejuvenation in the elderly focuses on counteracting the effects of immunosenescence and inflammaging, which are characterized by the decline in immune function and chronic low-grade inflammation. Therapeutic strategies include the use of mTOR inhibitors like rapamycin to enhance T-cell function and reduce inflammation, as well as cytokine and growth factor administration to boost immune cell activity, particularly macrophages and T cells. Stem cell therapies, such as induced pluripotent stem cells (iPSCs), and interventions targeting pathways like NF-κB, HIF-1α, and p38-MAPK are also explored to restore immune balance. Additionally, caloric restriction and functional foods that activate anti-inflammatory pathways, such as Nrf2 and NF-κB, offer non-pharmacological approaches to support immune system rejuvenation [53]. 

In a recent study, researchers developed the inflammatory clock of aging (iAge, Stanford University and Buck Institute for Research on Aging, Stanford, CA, USA) using deep learning on immune biomarker data from 1001 individuals aged 8–96 years. The iAge score was linked to multimorbidity, frailty, cardiovascular aging, and exceptional longevity, particularly highlighting the chemokine CXCL9 as a significant contributor to age-related inflammation. CXCL9 was shown to promote endothelial cell senescence, impair vascular function, and was strongly associated with adverse cardiac remodeling and arterial stiffness. A regression analysis found iAge to be predictive of frailty seven years in advance, with a correlation coefficient of R^2^ = 0.81 (*p* < 0.001). Further validation in healthy older adults confirmed that higher CXCL9 levels correlated with cardiovascular aging markers, such as increased pulse wave velocity (R = 0.22, *p* < 0.01). These findings suggest iAge is a potentially valuable tool for predicting age-related health decline and identifying potential therapeutic targets like CXCL9 to mitigate inflammaging [54].

### 4.3. Development of Novel Antibiotics

Antibiotic resistance is a significant challenge in elderly populations, particularly with the rise of MDR bacteria such as *K. pneumoniae* and *A. baumannii*. To combat these pathogens, ML can significantly accelerate antibiotic discovery. ML models can assist research in the analysis of vast chemical spaces to uncover potential antibiotic candidates that traditional methods might overlook. A prime example is the discovery of Abaucin, a novel antibiotic specifically targeting MDR *A. baumannii*. ML, using neural network models, was key in screening nearly 7500 molecules to identify this compound, which inhibits bacterial growth and shows promise in preclinical testing. Additionally, explainable deep learning approaches, such as graph neural networks, can potentially be utilized to predict both antibiotic activity and cytotoxicity across millions of compounds [55]. 

### 4.4. Antivirulence Therapies

Antivirulence therapies represent an innovative approach to combating bacterial infections by targeting the mechanisms pathogens use to cause disease rather than killing the bacteria directly. This strategy reduces the selective pressure for resistance development, a key issue with traditional antibiotics. By focusing on disabling virulence factors like adhesins, toxins, biofilm formation, and quorum sensing, antivirulence drugs can weaken bacterial pathogenicity without exerting the same evolutionary pressure that drives the emergence of resistant strains. Studies have shown that these therapies can be especially effective against MDR pathogens, offering a potential alternative or complement to conventional antibiotics, particularly in the treatment of chronic and nosocomial infections. While promising, antivirulence therapies still face challenges in terms of fully clearing infections, especially in immunocompromised patients, which may require combination with traditional antibiotics. Future research is likely to focus on enhancing the efficacy of these therapies and overcoming clinical challenges [56].

### 4.5. Monoclonal Antibodies

In addition to vaccination strategies and novel antibiotics, monoclonal antibody therapies offer a promising avenue for secondary prevention of bacterial infections, especially in elderly patients who are particularly susceptible to recurrent infections. One such example is Bezlotoxumab, a monoclonal antibody that specifically targets toxin B, the primary virulence factor of *C. difficile*. This antibody, considered an antivirulence therapy, has been shown to significantly reduce the risk of recurrent *C. difficile* infections, which are particularly dangerous in older adults with weakened immune systems or those recovering from antibiotic therapy. Bezlotoxumab is administered alongside standard antibiotic treatments for *C. difficile* and works by neutralizing toxin B, thereby preventing the damage it causes to the colonic mucosa. Clinical trials have demonstrated its effectiveness in reducing the recurrence of *C. difficile* infection, making it an important tool in secondary prevention, particularly for high-risk elderly patients who are prone to recurrence due to immunosenescence and microbiome disruption [57]. 

Looking ahead, there is significant potential for the development of similar monoclonal antibodies targeting other bacterial pathogens that disproportionately affect elderly populations. These therapies could be designed to neutralize specific bacterial virulence factors such as toxins, adhesins, or biofilm-forming proteins, providing targeted protection against secondary infections. For example, monoclonal antibodies targeting *S. aureus* toxins or *K. pneumoniae* adhesins could be used to prevent recurrent skin infections, bloodstream infections, or pneumonia in elderly patients [58,59]. 

### 4.6. ML for Mapping Bacterial Virulence 

Antivirulence strategies targeting virulence factors have gained attention as an alternative to traditional antibiotics, especially given the rise in antibiotic resistance. To support these efforts, the accurate identification and prediction of virulence factors are essential. Most computational models for virulence factor identification rely primarily on amino acid sequences, overlooking the structural information of proteins. To address this gap, a new approach, GTAE-VF (Graph Transformer Autoencoder for VF identification), has been introduced. GTAE-VF leverages 3D protein structures predicted by ESMFold and transforms the virulence factor identification task into a graph-level prediction problem. This model integrates a graph convolutional network (GCN) with a transformer, allowing for all-pair message passing to capture both local and global information. The decoder reconstructs the learned representations, which are subsequently used for virulence factor prediction. GTAE-VF has demonstrated superior performance in experiments, achieving an AUC of 0.963, outperforming other structure- and sequence-based models. The model’s ability to incorporate both structural and sequential data provides a significant advantage in predicting VFs, making it a potentially valuable tool for designing antivirulence strategies [60].

In a similar vein, a recent study constructed a large dataset of 3446 virulence factor classes with over 160,000 sequences and developed a deep convolutional neural network (CNN) model, VFNet (Multimedia Laboratory, Chinese University of Hong Kong, Hong Kong, China), to classify these virulence factors. VFNet automatically learns feature representations from raw sequence data and achieves high accuracy (0.9831) and F1-score (0.9803) for common virulence factor classes with sufficient samples. For uncommon virulence factor classes with limited samples, VFNet learns transferable features from auxiliary data, achieving accuracy from 0.9277 to 0.9512 and F1-scores from 0.9168 to 0.9446, outperforming traditional classifiers by 1–13% in accuracy and 1–16% in F1-score. VFNet’s ability to combine automatically learned and predefined features significantly enhances performance, especially for uncommon virulence factor classes. The model’s ability to detect conserved motifs further supports its effectiveness, making it a potentially useful tool for future virulence factor classification and antivirulence strategies [61].

### 4.7. ML in Predicting Antimicrobial Resistance and Empirical Therapy Selection

Traditional susceptibility tests often delay effective treatment, necessitating empirical antibiotic therapy, which may be suboptimal. ML could potentially offer a solution by predicting resistance patterns, enabling earlier, more targeted treatments. Recent studies in ICU settings have demonstrated the potential of ML algorithms—such as support vector machines, random forests, and multilayer perceptron—to predict antibiotic resistance using basic data like Gram stains, sample type, and patient demographics. These models achieved accuracies of up to 72.6%, providing crucial insights before lab results were available. Additionally, AutoML platforms like Microsoft Azure AutoML (Microsoft, Redmond, WA, USA) have shown promise in automating the model selection process. In one study, a stack ensemble model, trained on 11,496 instances, achieved a high area under the curve score of 0.850, significantly improving predictions when using the Synthetic Minority Oversampling Technique to balance data. ML can help clinicians in selecting appropriate empirical therapies, particularly for pathogens like *K. pneumoniae* and *P. aeruginosa*, reducing unnecessary broad-spectrum antibiotic use. As ML tools evolve, integrating patient-specific clinical and genetic data could further enhance their predictive power, enabling more personalized and precise treatments for elderly patients at high risk of infection-related complications [62,63,64].

### 4.8. Application of ML in Understanding Bacterial Virulence and Fitness Factors

ML models can potentially be designed to analyze bacterial genomic, proteomic, and metabolic data to distinguish virulence factors from fitness factors. By training ML algorithms on large datasets of bacterial genes and proteins from both pathogenic and environmental contexts, ML can identify patterns and associations that reveal whether a gene product’s primary function is to promote survival (e.g., siderophores for iron acquisition) or to evade the host immune system (e.g., capsules that prevent phagocytosis). For instance, unsupervised learning techniques like clustering could be used to group bacterial factors based on their expression under different environmental pressures (e.g., host vs. non-host environments), highlighting those that are more active in host-specific conditions as potential virulence factors [65,66]. 

Additionally, supervised learning models could be trained using annotated datasets where factors are pre-labeled as either virulence or fitness factors. These models can then predict the function of uncharacterized gene products based on features such as structure, homology, or interaction with host pathways. This could allow ML to predict which bacterial mechanisms—such as secretion systems—serve dual roles: originally evolving to protect bacteria from protist predation but later adapted to enable human infection. In combination, reinforcement learning could further optimize therapeutic strategies by simulating how targeting fitness or virulence factors affects bacterial survival, guiding the development of more precise antimicrobial treatments [65,66].

## 5. Research Gaps and Future Directions

Despite significant advancements in understanding immunosenescence and bacterial virulence, several research gaps remain. First, the complex interplay between immunosenescence and bacterial virulence factors, such as biofilm formation and quorum sensing, requires further elucidation. For example, the mechanisms by which specific bacterial strains exploit the weakened immune system in aging individuals, especially in relation to immune evasion and biofilm persistence, remain poorly understood [67]. Additionally, while ML techniques such as GNNs have shown promise in modeling bacterial-host interactions, more research is needed to integrate ML models with real-world clinical data to predict outcomes and optimize personalized interventions for the elderly. Finally, the limited efficacy of vaccines in aging populations underscores the need for research focused on identifying novel adjuvants or therapeutic strategies that can overcome the age-related decline in immune responsiveness [68]. 

Future research should focus on developing novel therapeutic interventions that target both immunosenescence and bacterial virulence mechanisms. For instance, emerging strategies such as antivirulence therapies, which inhibit specific bacterial virulence factors without exerting selective pressure for resistance, could be combined with immune-boosting agents to enhance the host’s defense against bacterial infections [69]. Furthermore, advances in ML, especially in GNNs and hybrid models, offer potential for personalized medicine approaches, allowing the identification of biomarkers that predict susceptibility to specific bacterial infections and responses to therapies in elderly patients [70]. Research should also prioritize the development of next-generation vaccines tailored for aging populations, incorporating ML-based insights to optimize antigen selection and adjuvant use [71]. Additionally, monoclonal antibodies targeting bacterial virulence factors, such as those used for *C. difficile*, represent a promising area for preventing recurrent infections in the elderly [72]. Finally, functional hypogonadism in elderly males has been associated with immunosenescence, and testosterone replacement therapy is another promising intervention that should be investigated in future studies [73]. Continued exploration of these innovative approaches could transform the management of bacterial infections in aging populations, improving both survival and quality of life. 

## 6. Conclusions

This review has highlighted the significant impact of immunosenescence on the susceptibility of elderly individuals to bacterial infections. Aging leads to a decline in both innate and adaptive immune responses, which is exacerbated by chronic inflammation, known as inflammaging. This weakened immune state allows bacterial pathogens to exploit immune dysfunction through various virulence factors, such as biofilm formation, capsule production, and immune evasion strategies. The prevalence of MDR bacteria further complicates treatment efforts, making elderly populations particularly vulnerable. ML technologies offer promising tools for mapping the complex interactions between immunosenescence and bacterial virulence factors, potentially leading to more effective vaccines, antibiotics, and personalized therapies. 

As the global population continues to age, it is imperative that healthcare systems adopt tailored approaches to manage bacterial infections in elderly individuals. Immunosenescence, coupled with the rise of MDR bacterial strains, creates unique vulnerabilities that demand personalized interventions. Future research should focus on developing advanced diagnostics, vaccines, and therapies that specifically address the immune deficits in this population. By leveraging ML and precision medicine, we can potentially move toward more effective prevention and treatment strategies, ultimately improving outcomes for aging populations worldwide. 

## Figures and Tables

**Table 1:** Mechanisms of immunosenescence and key bacteria involved.

Mechanisms	Description	Key Bacteria Involved	References
Thymic involution and T cell dysfunction	Thymic atrophy reduces the output of naive T cells, diminishing the immune system’s ability to respond to novel antigens. Over time, the T cell repertoire becomes restricted, with an increase in memory T cells, many of which may be senescent, expressing less of the co-stimulatory molecule CD28.	*M. tuberculosis*, *Listeria monocytogenes*, *Legionella pneumophila*, *Salmonella* spp., *P. aeruginosa*, *S. aureus*	[13,14,15,16]
Impact on T helper cells	Aging affects CD4+ T helper cell functionality, disrupting the balance between Th1 and Th2 responses. There is a shift towards Th2 dominance, which can compromise the clearance of intracellular pathogens and alter inflammatory responses, while regulatory T cells show reduced suppressive functions.	*M. tuberculosis*, *Listeria monocytogenes*, *Legionella pneumophila*, *Salmonella* spp., *P. aeruginosa*, *S. aureus*	[13,14,15,17]
B cell dysfunction	Age-related decline in B cell lymphopoiesis, altered signal transduction, and decreased production of high-affinity antibodies, affecting both vaccine responses and susceptibility to infections.	Encapsulated bacteria (e.g., *S. pneumoniae*, *H. influenzae*, *N. meningitidis*)	[13,14,15,18]
Senescence-associated secretory phenotype (SASP) and inflammaging	Senescent immune cells accumulate, secreting pro-inflammatory cytokines that contribute to a state of chronic low-grade inflammation, exacerbating tissue damage and potentially promoting the progression of age-related diseases. This environment can aid certain bacteria in evading immune surveillance.	*Enterobacterales*, *C. difficile*, *Legionella pneumophila*, *S. aureus*, *P. aeruginosa*	[13,14,15,19]
Innate immune dysfunction	Aging leads to impaired function of innate immune cells such as neutrophils and macrophages. These cells show reduced phagocytosis, chemotaxis, and cytokine production, making them less effective in managing bacterial invasions.	Most pathogens	[13,14,15,20]

## Data Availability

Data sharing is not applicable.
